# The Perils of Predatory Journals and Conferences

**DOI:** 10.5704/MOJ.2007.003

**Published:** 2020-07

**Authors:** S Ibrahim, A Saw

**Affiliations:** 1 Department of Orthopaedics and Traumatology, Universiti Kebangsaan Malaysia Medical Centre, Kuala Lumpur, Malaysia; 2 Department of Orthopaedic Surgery (NOCERAL), University Malaya Medical Centre, Kuala Lumpur, Malaysia

## Abstract

Predatory journals and conferences have little or no peer review. Their raison d'être is for making money through the article processing charges and the conference registration fees. Without a critical evaluation, predatory journals publishing flawed results and conclusions would cloud the existing scientific literature. Predatory conferences are the offshoots of predatory publishing. The conferences are not organised by learned societies, but by profit-making event organisers. There is a need for awareness among researchers and clinicians regarding predatory publishing. The scourge of predatory publishing and conferencing should be more often highlighted during scientific meetings and publication courses.

## Introduction

Have you ever received an email from an unknown sender inviting you to submit an article for an open access journal or to speak at a conference? If so, you are not alone. This is the modus operandi of these scammers - sending spam emails to unsuspecting researchers. Driven by the maxim of ‘publish and perish’, young researchers may unwittingly become victims of these scams. Predatory journals and conferences have little or no peer review^1, 2^. Their raison d'être is for making money through the article processing charges (APC) and the conference registration fees.

In the 2000s, funding bodies started encouraging researchers to publish in open-access journals^3^. Over the last decade, a group of profit-based open-access journals and publishers emerged to offer the opportunity to publish with high acceptance rates and short processing time (within days), without providing basic quality control as expected by the scientific community^4, 5^. Coupled with aggressive email soliciting, many young researchers were lured by these offers, without realising that there would be little or no peer review. Moreover, once the manuscript has been accepted, it would not be archived or indexed as promised^6^.

Jeffrey Beall first used the term “predatory” to describe these questionable journals / publishers^7^. Although some argued that his method of selection was too subjective and not validated, the “Beall’s list” of predatory journal /publishers has been helpful in guiding researchers and academics to address this serious issue^8, 9^.

These predatory journals may invite an author to submit papers in a field that is totally not related to the research area or experience. Occasionally an invitation may cite an article that the author had published in a legitimate journal^2, 10^. Mercier *et al* reported that over a period of 12 months, one recently graduated physician (who is also a research fellow) received a total of 502 unsolicited email invitations, in which 237 were for manuscript submission, 205 were for attending, speaking or organising a conference, 30 to serve in a journal editorial board, 6 to become guest editor of a special journal issue and one to be the editor-in-chief of a journal^11^. Similarly, an orthopaedic journal editor received 16 invitations to join editorial boards and 3 invitations to keynote conferences within a single day^12^.

### How Pervasive Is This Problem?

In 2012, Bohannon submitted a fictitious sting paper to selected online journals that were listed in Beall’s list and/or the Directory of Open Access Journal (DOAJ). Of the 304 journals, 98 (32.2%) accepted the paper^13^. When analysed based on publishers, 82% of those in Beall’s list accepted the seriously flawed article. Alarmingly, 45% of publishers listed in DOAJ also accepted the article. The DOAJ has since implemented more stringent selection criteria after March 2014, and re-evaluated previously listed journals^14, 15^. Another sting paper on mitochondria with “Star Wars” monologue as the content was accepted by 4 out of 9 biomedical journals ^16^. In 2015, Shen and Bjork reported that over 75% of the predatory journals in Beall’s list were operating from Asia and Africa^17^.

In 2018, Yan *et al* reported on 104 suspected publishers producing 225 predatory orthopaedic journals^18^. Only 82 orthopaedic journals were listed in the Journal Citation Report (JCR) by Thomson Reuters at the same time. Twenty journals were indexed in PubMed, and one journal was indexed in the DOAJ. The median APC for the predatory journals was US$ 420 (range 75 to 3,619) compared to US$ 2,900 (range 0 to 4,754) for legitimate journals. Most of the predatory journals were apparently located in the USA (56.4%), followed by India (13.8%) and the UK (6.7%). The true locations are probably unknown as many addresses are fictitious. The highest number of published articles were from India (2,353), followed by the USA (1,496) and the UK (717). Malaysia with 81 articles was the only nation from South-East Asia reported in this study.

Predatory conferences are the offshoots of predatory publishing. The conferences are not organised by learned societies, but by event organisers with the aim of profit-making. The name of the conference usually starts with “World, Global or International Conference of ….”, followed by a relatively general title^19, 20^. Some of the eminent faculties shown in the conference announcements may not be aware that their names were being used to promote these events. Another feature would be the lack of participation from national or regional faculty, no complimentary hospitality, and no honorarium even for the guest speakers^19^. In addition to the lack of vetting of the submitted abstracts, there would be many categories of awards or certificates offered to those who presented, usually with additional fees^21^. Others may be offered the opportunity to publish their work as conference proceedings in journals organized or owned by these event organisers^2^.

Even online meetings are not spared from exploitation by these profit-making organisers. The host may invite prospective speakers to register or present their topic through internet-based applications, and these will most likely lead to special awards or certifications^21^. Major cities in the Middle East and North Africa have recently become the epicentres of these predatory conferences^19^. Kuala Lumpur too has not been spared the scourge of the these organisers. In March 2017, the Malaysian Orthopaedic Association sent out an email alerting all members about an invitation from a questionable “Global Annual Meeting” for orthopaedic surgeons which was scheduled for July 2017.

### How Are We Affected?

Peer review is the hallmark of scientific publishing. Following critical review by scholars in the same field, new knowledge would be added into the literature to provide solution for existing problems or serve as stepping-stones for future advancements^2^. Without a critical evaluation, predatory journals would publish flawed results and conclusions that would potentially cloud the existing scientific literature or interfere with future studies including meta-analyses on these subjects^2, 10^.

In the field of health and medicine, poorly designed studies with faulty conclusions might be translated into wrong diagnosis and suboptimal treatment for some critical diseases. If an article reporting on an important scientific break-through or potentially life-saving discovery was submitted to a predatory journal, the material may be permanently lost because it would not be indexed by search engines or stored in archives or public repositories like PubMed Central (PMC)^1, 22^. Damage control by requesting for withdrawal or retraction is rarely successful, especially when a submission fee had been paid^23^. To make matters worse, it may not be possible to submit the material to other journals because this would be considered as a duplicate submission^1, 2^.

In addition, publishing in these journals may adversely affect the reputation of the author, institution and funding agencies. The career path of a junior researcher or academic may be blocked by inadvertent association with these journals^10^. It is not only authors from low and middle-income countries who are the main targets of these fraudulent publishers. It has been shown that many authors from high-income countries were also affected^24^.

Many research grants are supported by public donations. When the research outcomes are lost to these journals/publishers (not available in the scientific literature), future support to these institutions and funding agencies may be reduced. Another equally important adverse impact will be the loss of confidence in research institutions that may include medical centres^2^. The public will be more sceptical with the research outcomes and reluctant to volunteer for clinical studies.

The open-access model has been considered the future of scientific communication since it allows immediate and seamless transmission of information. PLoS One and many BMC journals are examples of open-access journals that have enriched the scientific literature. However, the emergence of predatory publishing would negatively impact these legitimate journals, especially new journals published by national or regional professional societies from low and middle-income countries. The decision by the Medical Council of India (MCI) not to recognise publications in all “e-journals” for the academic appointments or promotions in 2015 is the most glaring example^25^.

We cannot expect predatory conference organisers to focus on issues pertinent to global interest or relevant to public health, since the main interest of the organisers is to make a profit. The predatory conference would be competing with academic institutions and professional bodies for industry sponsorship and registration of participants^21^. It is not difficult to notice that usually these programmes and exhibitions are heavily influenced by one or two major industry sponsors^19^.

An over-emphasis of publications for academic and professional advancement is an important contributor to the emergence of predatory publishing and conferencing, especially in low and middle-income countries where English is not the first language^26^. In countries with limited staff and research facilities, stressing too much on research output in the form of publications may be counter-productive since precious time and efforts would be allocated to conducting low level research projects instead of focusing on training of the younger generation or resolving urgent problems faced by the community.

In some cases, the authors may not be as naïve as presumed. Due to pressure to publish, it has been shown that some authors intentionally submit to these questionable journals in order to improve their reputation or decorate their curriculum vitae^27, 28^. Whatever the reason, the action is not ethically and professionally acceptable. Other stakeholders from the medical industry may also collaborate with these publishers and event organisers to benefit from these practices^29^.

Some may question the appropriateness of the word “predatory”, since active promotion or advertisement of a publication or conference by itself is not unethical. It is the lack of critical evaluation and editorial support by qualified peers that contribute a breach of trust since this is expected by the scientific community. Other terms have been proposed as the alternative to “predatory”, but there is no consensus on this^2^.

### What Can We Do?

With the current pressure on junior researchers to publish, it will not be easy to eliminate this serious problem that challenges the reputation and legitimacy of scientific development. There are a few general approaches that we can consider.

There is a need for awareness among researchers and clinicians concerning predatory publishing. Predatory publishing should be more frequently discussed during scientific meetings, and it should be one of the main topics in science and biomedical publication courses. Creating awareness about the existence of these journals/publishers, and sharing information on their characteristics and common practices would be helpful. Authors should scrutinize the backgrounds of editorial members and verify the published journal metrics on legitimate websites. Some predatory journals use fabricated journal metrics such as the Journal Impact Factor (JIF), Universal Impact Factor (UIF), or Global Impact Factor (GIF) compiled by bogus companies^2^.

In 2013, the Committee on Publication Ethics (COPE), the DOAJ, the Open Access Scholarly Publishers Association (OASPA), and the World Association of Medical Editors (WAME) published the 16 principles of transparency and best practice to guide the medical / scientific community on how to identify legitimate publishers^30^ (Table I).

**Table I:** Warning signs of fake journals, based on the 16 principles of transparency^2^

Website: The journal’s website contains misleading or false information (e.g., indexing, metrics, membership of scholarly publishing organisations), lacks an ISSN or uses one that has already been assigned to another publication, mimics another journal/publisher’s site, or has no past or recent journal content. Name of journal: The journal’s name is the same as or easily confused with that of another. Peer review process: Peer review process and model are not mentioned. Manuscript acceptance or a very short peer review time is guaranteed. Submitted manuscripts receive inadequate or no peer review. Ownership and management: Information about the ownership and/or management is missing, unclear, misleading or false. Governing body: Information on the editorial board is missing, misleading, false, or inappropriate for the journal; full names and affiliations of editorial board members are missing. Editorial team/contact information: Full names and affiliations of the journal’s editor/s and full contact information for the editorial office are missing, the editor-in-chief is also the owner/publisher, or the editor-in-chief is also the editor of many other journals, especially in unrelated fields. Copyright and licensing: Policies and notices of copyright, publishing licence and user licence are missing or unclear. Author fees: Mandatory fees for publication are not stated or not explained clearly on the journal website, submission system, or the letter of acknowledgement and/or are revealed only in the acceptance letter, as a condition of acceptance. Process for identification of and dealing with allegations of research misconduct: There is no description on how cases of alleged misconduct are handled. Publication ethics: There are no policies on publishing ethics (e.g., authorship/contributorship, data sharing and reproducibility, intellectual property, ethical oversight, conflicts of interest, corrections/retractions) Publishing schedule: The periodicity of publication is not indicated and/or the publishing schedule appears erratic from the available journal content. Access: The way(s) in which content is available to readers, and any associated costs, is not stated, and in some cases listed articles are not available at all. Archiving: There is no electronic backup and preservation of access to journal content (despite such claims). Revenue sources: Business models, business partnerships/agreements, or revenue sources are not stated; publishing fees or waiver status are linked to editorial decision making. Advertising: Advertising policy is not given, or advertisements are linked to editorial decision making or are integrated with published content. Direct marketing: Direct marketing is obtrusive and gives misleading or false information.

Some institutions and professional societies have decided to develop blacklists of journals related to their fields of research. More recently, authors now rely on a “white list”, which are the lists of journals included by organizations like the DOAJ^31^, Scimago Journal Ranking (SJR)^32^ or Journal Citation Report^33^ to identify legitimate biomedical journals before submitting their scientific work.

Since November 2014, the DOAJ has adopted more stringent selection criteria to include new journals. Journals that comply with the following seven conditions will be considered to receive the DOAJ seal to certify high publishing standards^10, 34^:

1. Uses DOIs as permanent identifiers.
2. Provides DOAJ with article metadata.
3. Deposits content with a long-term digital preservation or archiving program.
4. Embeds machine-readable CC licensing information in articles
5. Allows generous reuse and mixing of content, in accordance with a CC BY, CC BY-SA or CC BY-NC license.
6. Has a deposit policy registered with a deposit policy registry.
7. Allows the authors to hold the copyright without restrictions.

Another way to avoid predatory journals or conferences is to use the checklist provided by “Think. Check. Submit”^35^ or “Think. Check. Attend”^36^ programmes. These checklists will guide researchers to determine the authenticity of a journal or conference. In addition, researchers need to perform regular online searches to ensure that they are not listed as speakers or editors with these organisations. This is a form of identity theft that may affect the academic and professional integrity of those involved^2^.

Another method is to modify the general approach of professional development especially in low and middle-income countries. Academic institutions should have a practical and realistic approach in planning for the future. An over emphasis on research and publications may lead young researchers to publish in predatory journals. In 2013, the World Health Organization (WHO) launched the Global Index Medicus (GIM) to encourage national and regional journals from low and middle-income countries to archive and index scientific papers^37^. Higher impact journals may consider reducing the APC to encourage submissions of manuscripts from low and middle-income countries.

Legal action against these publishers for failure to provide services that they have advertised may be taken. Many predatory publishers and conference organisers are also involved in copyright and trademark infringements, plagiarism and others forms of unethical practices. Editors, publishers, funding agencies, academic institutions, professional societies and other stakeholders should take the challenge seriously because the unethical practices are bound to damage the credibility and legitimacy of evidence-based science. These practices should be criminalized using the court system^2^. However, it may be difficult for a scholar to bring a predatory publisher or event organiser to court when they are most likely operating from a non-existing platform.

Successful withdrawal of a paper submitted to a predatory journal has been reported^23^. In 2019, the US Federal Trade Commission won a legal case against the OMICS Group and their companies for deceptive claims for their journals and conferences with a fine of about US$ 50 million^28^. It is understandable that this approach may not be easy because some of the stakeholders have vested interest in the overall outcome from these fraudulent endeavours^38^.

## Conclusion

Scientific discoveries, creative innovations and medical breakthrough should be accurately communicated without undue delay for acceptance into the scientific literature or translation into clinical practice. Online publishing allows publishers to accept more articles with less cost, while the open access model allows rapid dissemination of scientific knowledge without barriers. Predatory journals and conferences are dangerous developments that may ruin scientific progress because they sacrifice quality for profit. Since the perpetrators do not adhere to accepted ethical principles, all stakeholders should adopt a multi-faceted approach to defend the integrity of evidence-based science before further harm occurs. National and international regulatory bodies have to take stern action to reject these predatory organizations.
